# Advances in Genomic Discovery and Implications for Personalized Prevention and Medicine: Estonia as Example

**DOI:** 10.3390/jpm11050358

**Published:** 2021-04-29

**Authors:** Bram Peter Prins, Liis Leitsalu, Katri Pärna, Krista Fischer, Andres Metspalu, Toomas Haller, Harold Snieder

**Affiliations:** 1 MRC/BHF Cardiovascular Epidemiology Unit, Department of Public Health and Primary Care, University of Cambridge, Cambridge CB1 8RN, UK; 2 Institute of Genomics, University of Tartu, 51010 Tartu, Estonia; Liis.Leitsalu@ut.ee (L.L.); k.parna@umcg.nl (K.P.); krista.fischer@ut.ee (K.F.); andres.metspalu@ut.ee (A.M.); toomas.haller@ut.ee (T.H.); 3 Department of Epidemiology, University of Groningen, University Medical Center Groningen, 9700 RB Groningen, The Netherlands; 4 Institute of Molecular and Cell Biology, University of Tartu, 51010 Tartu, Estonia; 5 Institute of Mathematics and Statistics, University of Tartu, 50409 Tartu, Estonia

**Keywords:** genetic discovery, genomic medicine, return of results, patient communication, biobank, Estonia

## Abstract

The current paradigm of personalized medicine envisages the use of genomic data to provide predictive information on the health course of an individual with the aim of prevention and individualized care. However, substantial efforts are required to realize the concept: enhanced genetic discoveries, translation into intervention strategies, and a systematic implementation in healthcare. Here we review how further genetic discoveries are improving personalized prediction and advance functional insights into the link between genetics and disease. In the second part we give our perspective on the way these advances in genomic research will transform the future of personalized prevention and medicine using Estonia as a primer.

## 1. Progress of Genomic Technologies Enabling Personalized Medicine

### 1.1. Overcoming the Limitations of Genetic Loci Discovery

#### 1.1.1. Power Gains Through Sample Size Increase

Traditionally, one of the simplest ways to uncover substantially larger parts of the so-called ‘missing heritability’—next to focusing on structural variants rather than single nucleotide polymorphisms (SNPs)—is by simply increasing sample in genomic discovery studies, which in turn increases statistical power [1]. From very early on, this has been done through combining genome wide association study (GWAS) results of individual studies in meta-analyses, facilitated by the formation of large GWAS consortia, which are often phenotype-specific. The recognition by public bodies of earlier successes in genetic discoveries through consortia and the importance of data-driven healthcare, led to the creation of several biobanks, with the first initiatives having been established over 20 years ago [2,3]. Currently, one of the most well-known public biobanks is the UK Biobank effort, that aims to improve prevention, diagnosis, and treatment of illness, and the promotion of health through society [4]. Up to now, roughly 500,000 samples have been collected with both genetic data and a wide range of phenotypical measurements, which are available for a small fee, to the wider scientific community in its entirety. Similar large public efforts are Lifelines in the Netherlands [5] and the Estonian Biobank [6]. An interesting source of biobank data comes from personal genomics companies such as 23andMe [7] where participants can opt to make their genomes available (anonymized) to the scientific community, combined with data retrieved from self-reported health surveys. As of 2020 23andMe had reached a 12 million customers milestone, what makes it one of the largest genetic research databases in the world.

#### 1.1.2. Public Availability of GWAS (Results) Data

Significant increases in statistical power are furthermore facilitated by utilizing publicly available data [8]. Large groundbreaking projects funded by the public such as the Human Genome project typically made their findings publicly available. For institutional projects (i.e., within non-governmental organizations/universities), however, retaining the data privately in order to maximize the generation of scientific publications and authorships has historically been the rule rather than the exception. There is a trend toward more openly sharing data with researchers and the public [9,10,11]. The Wellcome Trust Case Control Consortium (WTCCC) study on seven common diseases [12] was a frontrunner in this regard, making the best use of often publicly funded research. This is exactly why platforms such as the database of Genotypes and Phenotypes [13] or the European Genome Phenome (EGA) [14] archives have been set up: facilitating easy data access and sharing. The volume of data from GWAS from various consortia presents logistical challenges in sharing data, but summary statistics from many of these studies are now available publicly, for example, through the OpenGWAS project (https://gwas.mrcieu.ac.uk/ (accessed on 29 April 2021)). This allows easy combination of study statistics or replication, and can be done even in the case of unknown proportions of sample overlaps by methods such as METACARPA [15]. It further enables downstream analyses using GWAS summary statistics only, such as large-scale Mendelian Randomization-like analyses using platforms such as MR-Base [16], or LD score regressions using LD hub [17]. To ensure that the vast amounts of generated data are not only publicly available, but also are truly accessible, other models to improve the portability of data are being developed, such as methods for decentralized storage of scientific data [18], and cloud-based models of multi-center collaborations [19].

#### 1.1.3. Increase in Resolution

##### Population Sequencing

Regardless of the sample size, genomic discoveries are dependent on variants that have been identified through sequencing. As we continue to sequence many more individuals, more variation is identified, and it has become clear that rarer variation may explain parts of the missing heritability for complex traits [20]. Therefore, improving genomic resolution is another feasible way to improve genomic discoveries, as has already been demonstrated early on by comparing HapMap to 1000 Genomes imputation for various traits and the observed improvement in terms of additional loci discovered and fine-mapping [21]. Sequence efforts continue to grow and projects such as the Haplotype Reference Consortium [22], which combine sequencing data from multiple cohorts, provide greatly enriched imputation panels, the creation of which will substantially improve genetic discoveries. A great number of population level whole genome sequence efforts are underway, such as in the UK (*n* = 500,000 [23]), Australia (*n* = 200,000 [24]), France (*n* = 235,000 [25]), and the USA (*n* = 1,000,000 [26]). Similar efforts to map and collect human genetic variation are ongoing for non-European populations, such as the African Genome Variation Project [27], H3Africa [28], Genome-Asia [29]. Multiple private sequence efforts are also underway, one of the most ambitious being the effort of the AstraZeneca to sequence 2 million human genomes [30], complemented by projects that will establish deeper and more accurate reference maps of the human genome [31].

##### Addressing Neglected Parts of the Genome

Even though many genomes have been sequenced and analyzed, certain regions in the genome are largely neglected in genome-wide analyses, such as the Y-chromosome or mitochondrial chromosomes. This is often due to complications in genotype calling, imputation, and selection of test statistics, as well as a lower proportion of genes, and a lower coverage of current genotyping platforms compared with autosomal chromosomes [32,33,34]. There is ample evidence for genetic regions in these chromosomes to be involved in a wide range of traits and diseases, including auto-immune diseases [35,36,37]. Investing in the large-scale analyses of these chromosomes, through efforts such as YGEN, the first international consortium that will assess the influence of Y-chromosome variation on complex traits of public health or evolutionary interest, are likely to provide a multitude of additional insights into the contribution of variants on neglected chromosomes to complex traits [38]. Moreover, mitochondrial work at scale is currently undertaken [39]. In a similar way, certain genetic regions have been rarely analyzed due to the difficulty to type and impute these regions, amongst the best-known examples immunologically are important complex regions such as the HLA region and killer cell immunoglobulin-like receptors (KIRs). Recent methods have however enabled the proper imputation of these regions [40,41], allowing the detailed investigation of their role in human disease.

##### Fine-Mapping

With much higher resolution genomic data as compared to traditionally imputed GWAS chip data, it becomes more likely that a causal variant will be present in the set of analyzed variants. However, it remains difficult to identify these, because the vast majority of associated variants fall outside the coding regions as recognized early on [42], whereas the majority of annotation efforts have traditionally focused on coding variants [43]. Developments in fine-mapping approaches such as CADD [44] and Eigen [45] have integrated extensive functional genomic annotations into a per-variant score of functionality, including conservation scores, epigenomic annotations and protein-level consequence scores. This importantly enables the prioritization of variants that are intergenic or in the non-coding regions, where previously this was challenging. Other approaches such as CAVIARDB [46], are able to highlight the most likely causal variants in a locus, just using summary statistics and PICS [47], provides similar fine-mapping capabilities, but then also includes prior knowledge such as transcriptional and epigenomic data, promising much improved fine-mapping results. Further fine-mapping opportunities lie in genetic diversity between populations, where differences in LD structure may help to further narrow down genetic regions associated with a trait [48], thereby more accurately pinpointing the location of actual causal variants. Studying different populations can improve the discovery of rare risk variants in loci already highlighted by common variants found by GWAS, as has been demonstrated in, for example, Greek isolates [49], or the Icelandic population [50].

#### 1.1.4. Increase in Throughput and Moving towards ‘Big Data’

Genetic discovery can thus be greatly aided by increasing sample sizes in combination with high-depth sequencing. This still comes at a substantial price, although the costs of whole genome sequencing have been rapidly declining, nearing the USD 1000 dollar genome barrier [51]. Newer developments offer further promise. One such technique is nanopore or ‘strand sequencing’ which allow sequencing of longer reads and faster phasing of genomes [52]. In concert with ever-increasing biobanking efforts are resulting in large amounts of data for which need to be matched by raw computational power and infrastructure. The quantity of data to organize and interpret has posed challenges, requiring the development of appropriate tools to address these issues. On the genomics analyses front, developments like Genotype Query Tools [53], provide novel data indexing strategies and much improved data compression, resulting in substantial query analysis performance improvements over current state-of-the-art tools of over 400-fold. In parallel, analytical algorithms are continuously optimized, whereby borrowing techniques from other fields of research such as signal processing [54] or artificial intelligence [55] offer further improvements in deciphering the human genomic architecture.

Developments on the analytical infrastructure front continue to contribute to ever more efficient genetic discovery. For example, sequencing pipelines, typically using algorithms running on high-end computer clusters on general CPUs, are being integrated in processors themselves (i.e., ‘hard-coded’). The reconfigurable DRAGEN Bio-IT Processor, produced by Illumina [56], has hard-coded highly optimized algorithms for the full next generation sequencing (NGS) secondary analysis pipeline, which set world speed records for genomic data analysis [57].

Solving data processing problems in big data genomics requires supercomputing infrastructure and expertise, which are not always available in academic environments. In industry, genomic big data management is tackled in various ways. Advanced genomics companies make use of large-scale commercial cloud-computing infrastructures available such as Amazon Web Services (AWS) or Google Genomics, to process and analyze sequence data using clever open-source distributed tools such as Hadoop or Apache SPARK that bring the algorithms to the data versus the much slower opposite. With the ever increasing size of genomics data, big data approaches like the use of computing clouds and advanced analytics platforms are already being adopted by the scientific community [58,59], simply out of sheer necessity.

### 1.2. Increase in Understanding (from Genotype to Phenotype)

Discovery and interpretation of genetic trait variation requires insights from multiple layers of biological intermediates through which genetic loci exert their effects on the phenotype in order to be able to obtain causal and mechanistic insights. This means integrating genetic data with expression data (eQTL), regulatory elements and effectors, proteomes, metabolomes, and intermediate phenotypes, all of which are interacting biological levels that have their effects on the final outcome studied.

Large efforts have been undertaken to map epigenomic data, such as in the ENCODE [60] and Roadmap Epigenetics Project [61], Blueprint Epigenome [62], but also for the proteome (HPP) [63] data exists on a comparable large scale. Efforts such as the Human Cell Atlas [64] and HipSci [65], which enable the retrieval of these data simultaneously from pluripotent stem cells allow insights in developmental and differentiation mechanisms on a cellular level. However, another phenotypic level to be considered is the microbiome. Apart from more intuitive associations of the microbiome with disease, such as inflammatory bowel disease [66], there are also indications that it influences psychiatric [67] and cardiovascular outcomes [68], whereas the composition of the microbiome itself is also influenced by genetic variation of the host [69], which brings yet another dimension to integrative genotype–phenotype analyses. Various efforts have been undertaken to obtain, map and interpret the aforementioned data types.

Other efforts even aim to map entire organs, which have the potential to uncover genetic effects on a precise organic scale. Examples include the UK Digital Heart Project [70], having reconstituted entire human hearts from echocardiographs or ENIGMA [71] with the world’s largest set of brain scans. Linking and integrating these biological data types will on the one hand undoubtedly improve our understanding of higher-order networks and mechanisms driving complex disease phenotypes across multiple tissues, but also bring along a great challenge to build statistical models, although promising methods are already available [72]. Many of the “omics” data can be readily integrated with genetic data using imputation methods. For example, epigenomic markers [73] or expression levels [74] that can be predicted based on cohort genotypes only. Imputation of epigenomes is useful as trait-associated variants affect regulatory regions in a cell-type dependent manner [75], and it is not always feasible to map every epigenetic mark in every tissue, cell type and condition of interest. Imputation of epigenomes for specific cell-types enables cell-type/tissue specific follow-up analyses to understand phenotype specific regulatory consequences of variants identified. Similarly, eQTLs can be cell-type specific [76,77], and appropriate cellular context enables a better understanding of variant consequences on expression level in relevant tissue.

### 1.3. Impact of Genetic Discoveries and Clinical Relevance

Translation of identified genetic variation or loci into pathogenic molecular mechanisms appears more feasible than ever before and allow for the realization of personalized medicine in practice. The need for such approaches is clear in clinical practice where the marked differences in response to therapies between different individuals are a recognized phenomenon, with drug response variability as a particularly well-studied case [78]. Medical conditions are personally unique when viewed at the molecular level; therefore, it only makes sense if treatment options follow suit. Generic treatment options can potentially have harmful consequences when “one size fits all” approaches are taken as evident from a wealth of drug response studies. As of today, GWAS findings have already proven to be clinically informative in a number of ways [79,80].

#### 1.3.1. Risk Prediction and Causal Inference

A currently more directly applicable use of genomic discoveries is (genetic) risk prediction of disease, where individual-level risk estimates through genomic risk scores (GRS) may help in early intervention and improve diagnostic procedures. There have been numerous studies developing risk prediction models using genetic markers and a number of successful examples [81,82]. Notably, a recent study using nearly two million genetic variants allowed stratification of individuals with different trajectories of CAD solely based on genetic information, and performed better than conventional risk factors at predicting incidence CAD [83]. With up to 2 million variants as a model for the genetic architecture of CAD, it is virtually impossible to pinpoint specific causal mechanisms to be used for intervention. Nevertheless, it is possible to perform causal inference using genetic risk scores through Mendelian randomization (MR) analyses. In this specific use of genetic risk scores, care must be taken however to design a risk score that instruments a particular exposure that is hypothesized to impose a causal effect on the outcome. For example, as previously suspected, it has been confirmed that LDL levels are causal to CAD [84], stressing that LDL-cholesterol lowering interventions such as adjusted diets and LDL lowering medication will have effects, as demonstrated also in a recent meta-analysis of randomized, controlled trials (RCTs) [85]. Similar approaches will allow the discovery of previously unknown causal risk factors that have the potential to aid disease management.

#### 1.3.2. Disease Stratification and Tailored Clinical Surveillance/Management

Another application comes in the form of disease classification, where for example Sirota and colleagues show that they were able to classify auto-immune diseases [86], identifying variants that make an individual susceptible to one class of autoimmune disease whilst simultaneously protecting from diseases in the other autoimmune class. This may enable clinicians to more optimally tailor treatment, as certain drugs for example are known to improve one type of autoimmune disorder, whilst having negative effects on another. A good example for this is infliximab, which is an antibody that binds to TNF. Typically prescribed and working well for rheumatoid arthritis and ankylosing spondylitis [87,88], it however has no efficacy and sometimes even worsens the condition in individuals with other autoimmune diseases such as multiple sclerosis [89].

#### 1.3.3. Personalized Treatment

A major area where GWAS may play a role is in pharmacogenomics. As modern medication only recently appeared as an environmental factor, it will not have caused any negative evolutionary selection pressures on common variants associated with (severe) adverse drug reactions (ADRs). Successful discoveries related to inflammatory disorders include identification of loci for ADRs against Lumiracoxib [90], a drug that is prescribed for the treatment of osteoarthritis and RA, causing liver injury, and loci associated with ADRs against thiopurin [91,92], prescribed for autoimmune disorders such as Crohn’s disease and rheumatoid arthritis, causing leukopenia and pancreatitis. In addition to the discovery of genetic risk loci for ADRs, tailoring treatment doses of drugs depending on an individual’s genetic profile will also become valuable in clinical practice and has already been demonstrated to be feasible for asthma [93].

GWAS can also aid in identifying drug targets [94,95]. By making clever use of known well-established and up to date gene-drug target databases such as DrugBank [96], therapeutic target database (TTD) [97], and PharmGKB [98], one can overlap genes identified in loci in a meta-analysis, or their interacting genes [99] and filter out those that appear druggable for further study. As many of the compounds in these databases are already FDA approved, this creates a wealth of opportunities for drug repurposing and repositioning, bypassing the necessary lengthy and costly process of clinical safety trials.

## 2. Estonia as a Primer for Personalized Medicine

### 2.1. Favorable Circumstances

In all, genetic discoveries will continue to contribute in various ways to the realization of personalized medicine [100], whereby personal biomarker-specific profiles affected by genetic, clinical, and lifestyle factors are likely to play an important role [101] (Box 1). The recognition of both the importance and the feasibility of personalized medicine for common complex disorders for national public health has incentivized the Estonian government to start an ambitious biobanking initiative aiming to completely overhaul its health-care system based on biologically informed personalized care, of which the resulting precision medicine tools will be made available to everyone as part of the basic health insurance coverage.

Box 1 **The advent of personalized medicine.**

It has long been recognized that standardized medical treatments, so called “one-size-fits-all” approaches, may have serious limitations. The idea of personalized medicine is, therefore, not new, but can be seen as an ongoing development. Prior to the biotech revolution of the last decades, methods to determine detailed biological differences between humans and populations that could be utilized for optimizing healthcare were limited. Decoding human DNA through the completion of the Human Genome Project resulted in what can be regarded as the establishment of a blueprint for the construction, development, and maintenance of the human organism. The HapMap project that followed, made an inventory of DNA differences between individuals and provided the groundwork for starting to understand inter-individual differences in genomic risk for disease. This genomic data, in concert with other upcoming health data such as other omics measurements, electronic health records (EHRs), and smart wearable devices can ultimately be devised to develop personally optimized intervention strategies.

Genomic data in particular has been shown to be useful in guiding drug development. For example, lead drug developer Astra Zeneca showed that genetically informed decisions not only led to an increase in number of successful drug development projects, but also a reduced time to market [102]. Similarly, for personalized medicine, genetic profiling already helps optimizing drug dosage and avoiding adverse effects, such as done for S-methyltransferase (TPMT) genotype testing prior to administration of a class of drugs named thiopurines [103]. Additional genetic testing helps to select medication for individuals with specific genetic mutations, an example being melanoma patients with particular mutations in the BRAF gene developing resistance against the normally used single agent BRAF inhibitors, who display a better response with combined use of MEK inhibitors [104]. The advance of personalized medicine is demonstrated by the vast increase of approved medication entering European and American markets, which define it generally as a treatment that recommends or requires a biomarker pre-test to determine if a treatment is suitable for a patient. In Germany, for example, according to this definition, there were no personalized drugs on the market before 1996. The decade that followed, saw 10 oncological drugs becoming available as personalized medicine, after which this number shot up to 63 in 2019, and diversified over various disease classes [105].

Many nations have already launched initiatives to promote personalized medicine [106], but Estonia in particular has shown to be on the forefront through its unique nation-wide integrated approach, connecting a national biobank with electronic health records with the aim to incorporate it into general healthcare.

**Figure 1:**
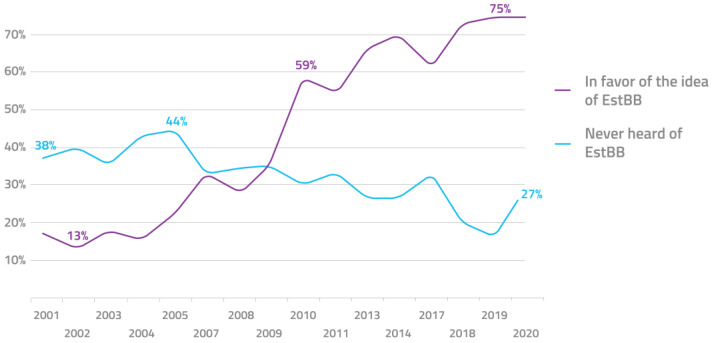
Public awareness and approval of the Estonian biobank over the past 2 decades.

This initiative is enabled by a number of aligning favorable circumstances. Firstly, the progressive political climate in Estonia and attitude of its population support the adaptation of new technological developments, including personalized medicine. The Estonian parliament was the first in the world to pass a special law to govern genome research in the general population, the Human Genes Research Act [107]. This law is favorable to the implementation of personalized medicine and provides the required legal framework for it. The various acting governments throughout the lifespan of the initiative have been overwhelmingly supportive of the initiative. Although, perhaps even more important in this context is the positive attitude of the general public. Regularly conducted surveys show that up to 75% of the respondents support the biobank, only 1% hold the opposing view, and around 77% are interested in genetic testing [108] (Figure 1).

Secondly, in the last decades Estonia has heavily invested in a first-class national IT infrastructure and know-how, which has digitized many aspects of daily life. At the center of this is the Estonian ID card—a computer-readable compulsory identification card implemented in 2002 that gives access to a nationwide governmental technical infrastructure called the X-road [109]. This is the central “digital highway” that gives access to all online services in the country. The X-road is highly adaptable in the sense that new functionalities can be added at any time. This includes the support for personalized medicine. The system can be used to link major Estonian health registries and hospitals and facilitate sharing of medical information for patients, such as health data history, hospital admissions, pharmacy prescriptions, to compile a full medical picture to which treatments can be tailored in a secure manner [110].

### 2.2. Biobank Cohort, Data Collected and Generated

The favorable circumstances culminated in the foundation of the National Biobank (“Estonian Biobank”, EstBB) in 2001, which was transferred to the University of Tartu as the Estonian Genome Center (EGCUT) in 2007. From 2002, it has built up its databases and research portfolio ever since [6,111]. By 2011, the population-based biobank had 52,000 adult participants from the first wave of recruitment, and in 2018–2019 an additional 150,000 participants were recruited in the second wave. Altogether the biobank cohort constitutes 20% of the country’s adult population. All projects described here are based on the first 52,000 subjects in the EstBB, not on the whole cohort of 202,000 as it is today.

Blood samples were collected from all participants, which were subsequently used for genotyping using the Illumina Infinium Global Screening Array, as well as for deep phenotyping. Whole genome sequence data is currently available for 3000 individuals, as well as whole exome sequencing for 2500 individuals and these numbers are continuously increasing. Furthermore, EstBB has measured transcriptomics (Illumina RNAseq), DNA methylation (Illumina 450 K methylation array), proteomics (Olink) as well as various metabolomics profiles (clinical biochemistry, NMR, mass spectrometry) for a large number of participants to generate precise information based on molecular profiles. In 2018, the EstBB launched a project for obtaining microbiome profiles from stool samples (over 2500 currently collected). Recently, a new contract was signed to expand the current Nightingale NMR metabolomics dataset (https://nightingalehealth.com/ (accessed on 14 April 2021)) to all biobank participants in 2021–2022. In combination with regular supplementation of electronic health status updates from registries, large scale longitudinal studies are facilitated.

### 2.3. Translational Research in Genomics

The Estonian biobank legislation and consent allow return of scientific results to biobank participants. The first projects involved a genotype first approach where biobank participants with specific genetic findings were invited for further phenotyping and clinical assessment while offering disclosure of genetic findings to the participant [112,113].

In 2017, a different approach was introduced offering a selection of results by the biobank to participants who expressed interest in receiving them. As part of the process, participants will update some measurements, update their lifestyle information and sign the consent for return of results all through an online participant portal. Over a two-year period, 2957 participants received the report accompanied with face-to-face counseling. On top of disease risk prediction, EstBB is analyzing the potential impact of considering pharmacogenomics in patient care [114]. This supports not only more tailored medication dosages, but also allows avoiding ADRs and medication that might not even be effective for a particular individual. Currently, the team at EstBB is able to profile 10 genes involved in compound metabolism to give feedback about 31 different medications.

Furthermore, the biobank has put an extensive monitoring practice in place to ensure and improve the quality of the entire process, with a focus on assessing the effect and outcomes of receiving genomic risk information. Through the participant portal, each participant is asked to fill out three questionnaires regarding the process: one before the return of results visit, one immediately after it, and a third 6 months later. The results of this pilot will inform future projects involving return of genomic risk information (manuscript in preparation). For instance, when is face-to-face counseling necessary and when would other modes, such as using the participant/patient portal for risk communication, be sufficient?

The most important result of these early translational studies, however, was the subsequent decision of the Estonian government to invest substantially in order to increase the biobank fourfold, to 200,000 subjects, and have all participants genotyped using arrays, and sequence a subset of 3000 individuals to provide a population-based reference panel for imputation of the array data.

### 2.4. National Personalized Medicine Pilot Projects

**Figure 2:**
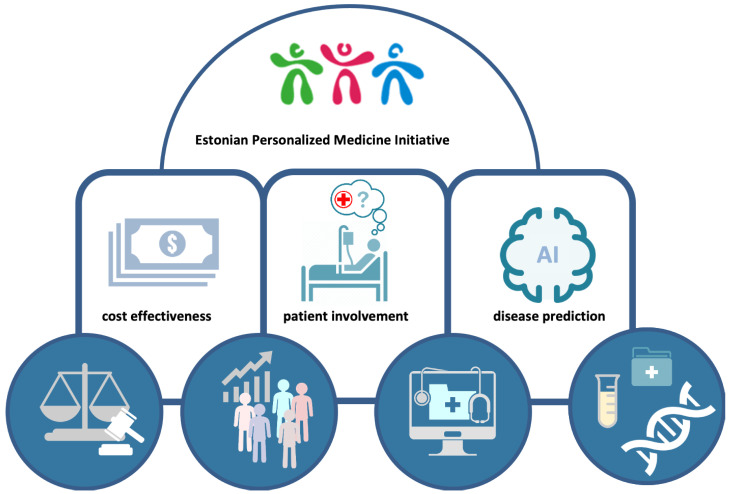
Implementation of personalized medicine. Four core elements (circles) that represent strategic advantages enabling the Estonian Personalized Medicine Initiative to be effectively realized.

While previous projects were conducted in a research context, these national personalized medicine pilots involve biobank participants as patients in clinical practice. To prepare for a nation-wide personalized medicine system (Figure 2), Estonia has just finished the two pilot projects involving participants of the EstBB, but conducted in the hospital settings and by hospital-based physicians and primary care providers (GP’s) (manuscript is in preparation). The main goals of these pilots were to test the feasibility of different approaches in clinical practice and demonstrate the benefits of incorporating molecular profiles in disease management in current clinical practice and in hospital IT structures.

Both of these national pilot projects involved 1000 biobank participants. The first project focused on personalized risk prediction and management of breast cancer and involves geneticists in combination with either primary care physicians or oncologists. The second project involved family physicians and focuses on CAD.

The Estonian Biobank research projects as well as the national personalized medicine clinical pilots are expected to lead to the gradual incorporation of personalized genome-based medicine into general health care in Estonia. The initial steps in this direction have been taken by the Ministry of Social Affairs of the Estonian Government when it announced the launch of the Personal Medicine Initiative in 2016 [115].

EstBB currently already actively participates in providing the genomic input both through genomic research and by providing the genotype information. Meanwhile other partners produced the necessary IT infrastructure changes to allow genomic data to be considered not separately but along with other health information, this includes pharmacogenomics integrated with the electronic prescription system, for instance.

**Figure 3:**
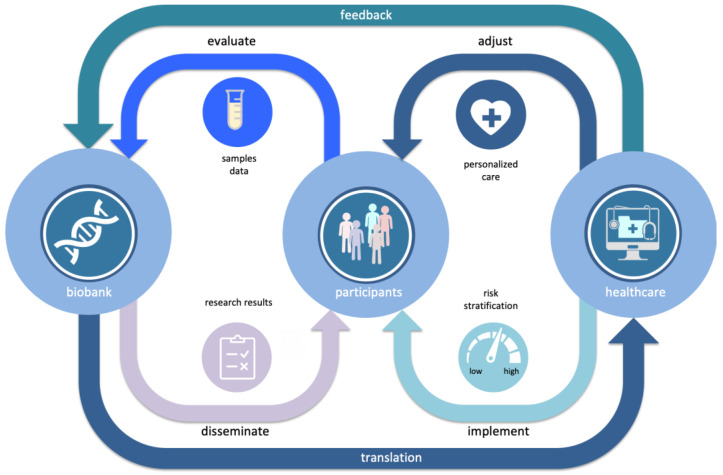
Learning healthcare cycle, where through continuous bi-directional interaction between a population biobank and health care systems new personalized genome-based information from biobank is gradually incorporated into the general health care.

As changes in medical care and health information of biobank participants is regularly updated the risk predictions can be improved as new data comes in (Figure 3).

## 3. Challenges and Pitfalls of Personalized Medicine Implementation in Estonia and Beyond

### 3.1. Scientific Challenges

The current pilot projects focus on tackling common complex diseases that have the highest burden in the nation and are known to have a substantial genetic basis, being type 2 diabetes, breast cancer, and CAD. One of the first major goals is to develop efficient predictors for the risk of each disease by integrating genetic, health, and environmental factors. The performance of the existing risk-prediction algorithms of the disease can be enhanced by the addition of newly identified genetic components, making the resulting risk estimates more personalized and accurate [116,117]. This in turn allows stratification of individuals into categories of different risk levels, allowing to differentiate between risk-reducing interventions targeted at those categories. Ultimately this will lead to an increase of cost-efficiency of interventions and an improvement of their efficacy.

In this context, however, one of the challenges for researchers is to develop an efficient genetic predictor capturing the polygenic nature of the heritable component of the disease. The traditional approach for developing genetic risk prediction algorithms encompasses the use of only a handful of genome-wide significant SNPs associated with a disease. The biostatistics team at the EstBB that focuses on GRS methodology has demonstrated that a polygenic risk score or ‘predictor’ that captures the effects of a large number of genetic variants in an additive manner, has good predictive ability [117], an approach now widely adopted [118]. Moreover, while conventionally the genetic variants are weighted by their estimated regression coefficients from GWAS, the EstBB biostatistics team has additionally demonstrated that further additional weighting can reduce biases in such weights caused by what is known in statistics as the “winner’s curse” phenomenon [119] and further improves the predictive accuracy. Such a doubly-weighted GRS has already been developed for type 2 diabetes, where it has been shown that the actual risk level for individuals with similar levels of conventional predictors (age, body mass index, physical activity, and diet) may differ by more than three times depending on the level of the GRS [117].

### 3.2. Data Security and Privacy Challenges

A major concern when conducting genetic research using the EstBB data and samples is honoring the need for privacy of the participants. Under no circumstances should the participant confidentiality be compromised unless he/she reveals it personally.

Most countries have no specific law that regulates usage of genomic data, thereby leaving a legal void that can lead to risky circumstances. For example, genetic information can be valuable for insurance or employment purposes, as genetic tests can be used to detect inherited conditions that might be costly to treat for the healthcare system, and put a burden on employers. This generates concerns regarding for example employment decisions, possibly the invocation of charging higher insurance premiums or even denial of coverage [120], fears that are not unreasonable [121]. These fears can have various consequences. Individuals denying genetic testing can miss out on health benefits these tests can facilitate. Lack of trust can also result in skepticism toward research in genomics and thereby result in decreased study participation, or even a reduction in funding.

Estonia, however, implemented a law in 2000 explicitly concerning the population biobank and genomics data [107]. Specifically, it regulates how genome data are allowed to be gathered and stored, to whom it belongs, and how it is allowed to be used in scientific and commercial research. The key principle is that the owner of the genomic data is always the individual. This is the basis of a robust system based on trust and transparency. All participant data is pseudonymized, and only a handful of individuals have access to the key that enables to link different types of data (phenotypes with genotype) [110]. Digital security is further enhanced by data storage on systems that are completely separate from the internet, making the system virtually unhackable from the outside.

### 3.3. Challenges in Communicating Research Results

Identifying high risk individuals early, focusing more on disease prevention, while also increasing patients’ participation and responsibility in taking care of their own health is expected to help decrease the burden of common complex disease in the long run. One important issue is the automated decision support algorithms and AI tools analyzing the vast and complex data and providing the report for the physician who has to communicate the content to the individual (who is or is not yet a patient). The complex nature of risk determination and stratification through genomic and phenotype data, as well as the probabilistic nature of the results are very likely to be beyond the grasp of the general population.

**Figure 4:**
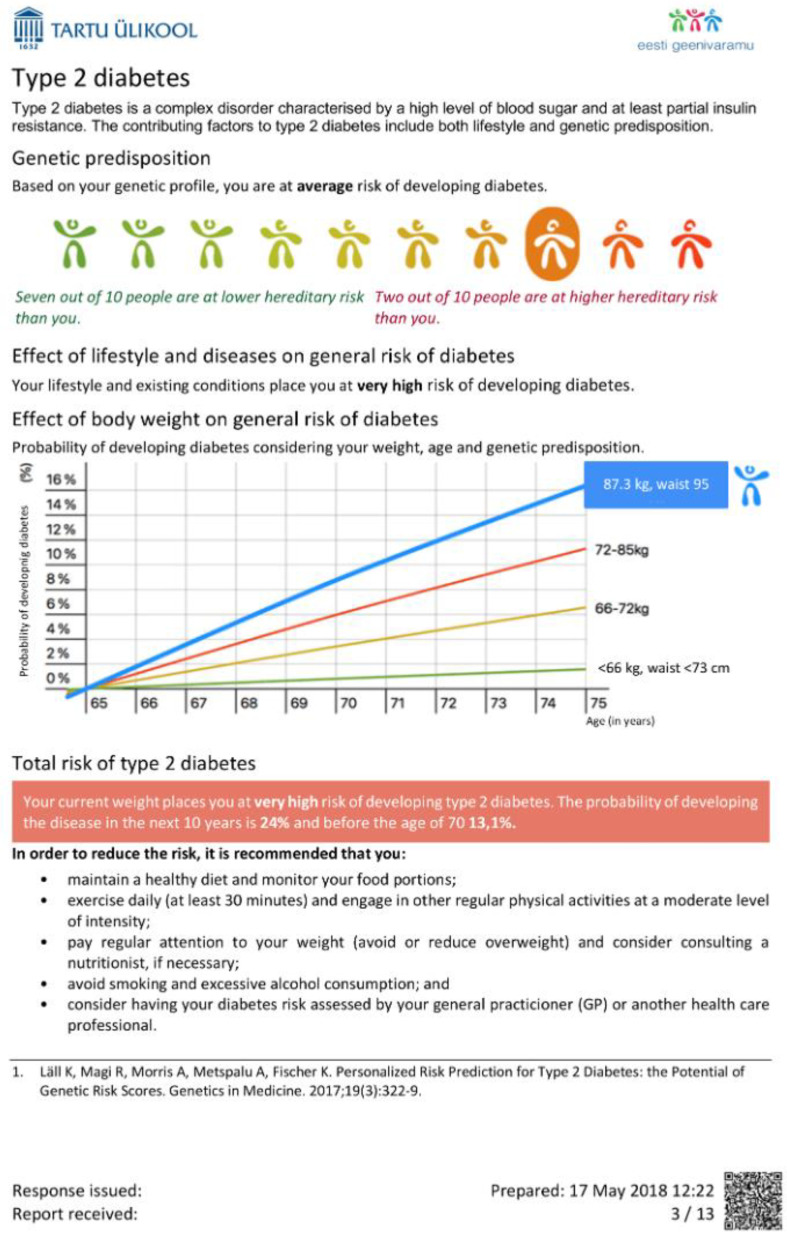
Example page in the concept report for an Estonian biobank participant. In a personalized report, the participant will receive information on how her/his risk of the disease will increase as a function of age, how much the genetic component has contributed to risk and to what extent it could be altered by lifestyle modifications or medical intervention.

Therefore, a challenge lies in the communication of these results, which are the aggregate of multiple layers of health-related information (genetic, clinical, psychological, etc.). To minimize the time spent on interpreting complex information, participants should have this information presented in an understandable format and language, with guiding visualizations and explanations (Figure 4 report example). Care must be taken that results are returned together with proper medical support to provide interpretation and translation, which includes genetic counseling, easy access to medical specialists and follow-up.

## 4. Future Outlook for Personalized Medicine in Estonia and Beyond

The EstBB project as well as the national personalized medicine clinical pilots are expected to lead to the gradual incorporation of personalized genome-based medicine into general health care in Estonia (Box 2). The initial steps in this direction have been taken by the Ministry of Social Affairs of the Estonian Government when it announced the launch of the national Personal Medicine Initiative in 2016 together with a long list of other stakeholders, including three other ministries, the main Estonian universities and hospitals, as well as private IT solution providers [115]. This project is led by the Institute for Health and Development and will end in 2023. It is currently developing the first three genetically informed decision tools to be used in clinical practice, which consist of polygenic risk scores for breast/ovarian cancer, CAD, and pharmacogenomic traits.

Box 2 **A future doctor visit.**

When the Estonian National Personalized Medicine Initiative is in full effect, the healthcare system in Estonia will be unlike any other system in the world. An individual born in 21st century Estonia, will have their genome sequenced in high resolution early in adult life, for perhaps no more than GBP 100. It then will be scanned for potential genomics risks for an array of common complex diseases, as well as more high-risk Mendelian disorders and polymorphisms causing adverse drug reactions. The results will be encrypted and stored in a secure database as part of the national biobank. These data are accessible for the individual, who retains ownership and absolute control over accessibility, but also for primary care and specialist physicians and pharmacists, on the condition that the owner provided access permission. Doctor visits and regular health checks populate the electronic health record database, and intelligent learning algorithms will then utilize this new information to improve disease prediction models. During visits, the GP will provide updated genomic risk profiles if needed, identify high risk lifestyle elements that need to be adjusted, and then will devise lifestyle advice and monitoring priorities based on this information. When medication is needed, potential suitable drugs for the treatment will first be cross-checked against a pharmacogenetic profile for potential adverse reactions and potentially needed dose-adjustments. The prescription is being prepared even before the participant leaves the consultation room, and upon arrival at the pharmacy, a national ID card is scanned and medication picked-up straightaway. Meanwhile, genomic and other health-related data is continuously growing and being used (anonymized) by international scientific consortia to identify previously unknown disease mutations and pharmacogenetically important genes. Once confirmed, these are fed back into the database, and individuals with high-risk mutations are informed and invited for a consult to provide suitable preventative care.

Some aspects of the national Personal Medicine Initiative already have tangible impact: at the moment 99% of health data has been digitized, 99% of prescriptions are digital, and 100% of healthcare billing is digital [122]. Moreover, specifically in terms of genomics-informed healthcare, selected findings in the framework of current pilot projects, such as pharmacogenetic profiles, high-risk mutations, interpretation of polygenic risk scores, are already being offered to involved participants. The national personalized medicine services are continuously further improved by, for example, adding and developing follow-up smart IT solutions such as clinical decision support software, but also increasing investments in health management and surveillance and education are being made.

As certain genetic discoveries continue to be made, along with an ever-increasing body of biological insights that are accepted and deposited in electronic health databases, the best practices in personalized medicine will change with time. As such it is key to periodically re-evaluate the medical risk information in light of newly accumulated knowledge and adjust intervention and communication strategies accordingly. Continued education and training of health professionals is also highly important so they are qualified to effectively apply genomic tests and communicate the garnered results to their patients. While family physicians are optimistic about envisioning genomics in their everyday practice they too recognize that additional training is necessary [123]. To prepare the physicians, genomic healthcare workshops are prepared, covering topics on genetic risk scores, pharmacogenomics, and genetic counselling. Over the next two years 900 medical professionals, mainly family physicians and nurses, will be able to participate. Additionally, the National Institute for Health and Development is sending out a monthly newsletter to keep health-care workers informed of developments in personalized medicine arena.

The potential of the Estonian personalized medicine initiative is boosted by the Estonian biobank with over 200,000 participants, the latter representing nearly 20% of the adult population. In the longer term the plan is to genotype most of the population, with a customized genotyping chip that includes Estonia-specific genetic variation, allowing the development of more precise genetic instruments for medical decision making. The European Union recognizes the need for knowledge transfer and supports these initiatives through ample funding in Horizon Europe programme from 2021 to 2027. Estonia is currently at the forefront of implementing eHealth services and genomic data in health care in the EU, and is sharing its knowledge on national digital healthcare and biobanking by participating actively in the EU Member States’ driven initiative “Towards access to at least 1 Million Genomes in the EU by 2022” [124].

Though the future of personalized medicine is looking bright, the universal key to the success of national personalized medicine projects will lie in education and demonstrating the public benefits of genomic screening, and an unrelenting effort to execute these efforts in a human rights framework that guarantee the ethical and secure use of data, and the privacy and ultimate ownership of participants.
